# Preliminary study of the production of metabolites from in vitro cultures of *C. ensiformis*

**DOI:** 10.1186/s12896-020-00642-x

**Published:** 2020-09-10

**Authors:** Juan F. Saldarriaga, Yuby Cruz, Julián E. López

**Affiliations:** 1 Dept. of Civil and Environmental Engineering, Universidad de los Andes, Carrera 1Este #19A-40, Bogotá, Colombia 111711; 2 Dept. of Environmental Engineering, Universidad de Medellín, Carrera 87 #30-65, Medellín, Colombia 050026

**Keywords:** *C. ensiformis*, pH, LED blue, LED red, Bioactive compounds, In vitro culture

## Abstract

**Background:**

*Canavalia ensiformis* is a legume native to Central and South America that has historically been a source of protein. Its main proteins, urease, and lectin have been extensively studied and are examples of bioactive compounds. In this work, the effect of pH and light effects on the growth of *C. ensiformis* were analyzed. Also, the bioactive compounds such as phenols, carotenoids, chlorophyll a/b, and the growth of callus biomass of *C. ensiformis* from the effect of different types of light treatments (red, blue and mixture) were evaluated. Likewise, the antioxidative activity of *C. ensiformis* extracts were studied and related to the production of bioactive compounds. For this, a culture of calluses obtained from seeds were carried out. For the light experiments, polypropylene boxes with red, blue, combination (1/3, 3/1 and 1/1 R-B, respectively) lights and white LED were used as control. In each treatment, three glass containers with 25 ml of MS salts containing 0.25 g of fresh callus were seeded.

**Results:**

The results have shown that the pH of the culture medium notably affects the increase in callogenic biomass. It shows that the pH of 5.5 shows better results in the callogenic growth of *C. ensiformis* with an average increase of 1.3051 g (198.04%), regarding the initial weight. It was found that the pH 5.5 and the 1/3 R-B LED combination had higher production of bioactive compounds and better antioxidant activity. At the same time, the red-light treatment was least effective.

**Conclusions:**

It was possible to find the ideal conditions of important growth under conditions of pH and light of *C. ensiformis*. Likewise, it is evaluated whether the production of compounds of interest, such as phenolic compounds and carotenoids, occurs under these conditions. The highest production of calluses occurs in the 1/3 R-B LED combined light treatment, which showed a significant increase in biomass, followed by B. From this study, it could be demonstrated that *C. ensiformis* produces compounds such as phenols and carotenoids in vitro culture that are essential for the antioxidant activity of the plant.

## Background

In biochemical research, *Canavalia ensiformis* has been historically a promising source of protein [1]. Urease and lectin from *C. ensiformis*, are widely studied proteins and notable examples of the importance of bioactive compounds of this plant species [2–4]. In plants, the amount of these compounds depends mostly on the plants growing conditions (in vivo or in vitro), on the photoperiod to which it is exposed and on other important factors such as planting density, nutrient supply, temperature, etc. [5]. Therefore, the absorption of nutrients in plants grown in vitro are affected by several factors, such as gelling agents [6], the breakdown of carbohydrates and chelating agents [7]. Furthermore, it has been reported that light is a factor that can affect the production of these compounds [8–10], thereby inducing physiological changes in plants [11].

Extracellular pH can affect ion absorption and, at the same time, creates ionic competitions [12]. Studies have shown that low pH levels are associated with inhibition of cation absorption, while anion uptake may be slightly influenced or not influenced [12]. In particular, attention has focused on the effect of pH on nitrogen uptake by roots and on how the predominant form of nitrogen (i.e., NH_4_^+^ or NO_3_) in the nutrient solution influences the absorption of the other ions [12–14].

Light is the origin of the direct source of energy for many plants as IT controls the growth rate in processes such as phototropism, photosynthesis, photomorphogenesis, among others that affect the metabolism related to pigments [15–17]. Four of the most important pigments are chlorophyll a (*Chla*), chlorophyll b (*Chlb*), phytochrome PR (red light) and PFR (far-red light), this according to Zhou et al. [16] and Wright [18]. These mainly absorb blue (400–500 nm), red (580–680), and far-red (690–800 nm) light. According to Carvalho et al. [19], the quality of these lights affects the accumulation of photosynthetic pigments in the leaves, which can increase absorption of light in low light conditions or act as screening pigments, and free radical scavengers in high light conditions. These effects have been extensively studied due these lengths are absorbed by photosynthetic pigments, and their important impact occurs in the development of plants [20, 21]. Studies have found that the wavelengths of R and B always coexist in natural light environments and that the optimal mixture of these differs from the plant species. For example, for the strawberry, a 7/3 ratio has been found [22]. In contrast, for rapeseed, a ratio 1/3 [23] of the RB mix has been reported.

Chen et al. [24], argue that blue light is necessary during plant growth for normal photosynthesis and that the regulated responses of this plant quantitatively resemble those of irradiation intensity. Also, it was shown that plants cultivated in B have a higher proportion of *Chl a/b*, more significant activity of Rubisco, and higher activity of transport of photosynthetic electrons than plants grown in R [25–28]. Also, B can trigger photomorphogenesis processes in plants and provides enough energy through photosynthesis to maintain normal growth and development [20]. Costa et al. [29] indicated that B is indispensable for photo-acclimatization and protection of diatoms at high light intensities.

On the other hand, plants that were grown with R exhibit a significantly lower *Chl a/b* ratio, lower rates of photosynthetic CO_2_ fixation, and total plant biomass than plants grown with white light or a combination of R and B [28, 30, 31]. According to Wang et al. [28], plants are sensitive to their light environment not only because the light is the sole source of energy but also due to its effect on growth and development. An example of the above takes place in autumn, spring, and winter, where the shortening of the light time and the considerable fluctuation of irradiation are quite serious problems for the development of plants [28]. Plants that grow under low light intensity are more sensitive to photoinhibition caused by exposure to increase light irradiation [32].

In vitro tissue culture has been widely used for rapid plant propagation and obtaining bioactive compounds from cell culture [33]. There are previous studies of a successful induction and propagation of calluses of *C. ensiformis* under in vitro conditions [34]. However, the production of bioactive compounds such as photosynthetic pigments, phenols, and carotenoids from cell culture has not been evidenced for *C. ensiformis*. The main objective of this work was to evaluate different types of pH of the culture medium to determine the optimum in which the planting of *C. ensiformis* achieves adequate growth, and the production of metabolites of interest that can be used in its crops.

Furthermore, starting from the optimal pH, the effect of different light treatments (red, blue, and combination), the production of chlorophyll, phenols, and carotenoids on the growth of callus biomass of *C. ensiformis* were evaluated. Also, the antioxidant activity of the extracts of *C. ensiformis* callus were analyzed, and these have related to the production of bioactive compounds. In this way, it was possible to determine under which light conditions, R, B, or combination present the best production of bioactive compounds and antioxidant activity.

## Results

The results have shown that the pH of the culture medium notably affects the increase in callogenic biomass. Table 1 shows that the pH of 5.5 presents better results in the callogenic growth of *C. ensiformis,* with an average increase of 1.3051 g (198.04%) after 30 days. While the pH of 6.0 evidences the least results of an increase in callogenic biomass. And finally, the pH of 5.0 and 5.7 show very similar results of biomass increase with approximately 104% on average for both treatments.

**Table 1:** Callogenic biomass variation of *C. ensiformis* at different cultivation pH

Factor	pH 4.5	pH 5.0	pH 5.5	pH 5.7	pH 6.0
Biomass initial weight (g)	0.7321	0.6294	0.6590	0.6113	0.6519
biomass final weight (g)	1.3708	1.1858	1.9641	1.2449	1.2814
average biomass increase (g)	0.6387	0.6564	1.3051	0.6336	0.6295
growth rate (%)	87.24	104.29	198.04	103.64	96.56

Table 2 shows the analysis of variance components for the effect of pH on callus growth, showing that pH is the most important factor in the effect of growth (*p*-value 0.005). It is observed as in the data in Table 1, that it is the pH of 5.5 that presents a positive effect on callus development. ANOVA shows that pH is the most significant factor and that it is important in callus growth. Of these, pH 5.5 is the one with the highest percentage increase since planting after 30 days of planting. While the pH of 6.0 and 4.5 are the treatments that show lower results of biomass increase. With these results, it is shown that the optimal growth for a significant increase of biomass in vitro cultures of *C. ensiformis*, is with a pH of 5.5.

**Table 2:** Analysis of variance for the effect of pH on growth

Source	Sum of squares	Df*	Mean square	***p-value***
Total (corr.)	256.6962	286		
pH	16.6853	4	0.8177	0.0056
Biomass initial weight	240.0109	282	0.7123	0.0676

*Df: degrees of freedom.*

From these tests, the effect of the growth of calluses of *C. ensiformis* were evaluated in a medium with pH 5.5 but with variation in the intensity of light.

### Effect of light on callus growth

**Fig. 1:**
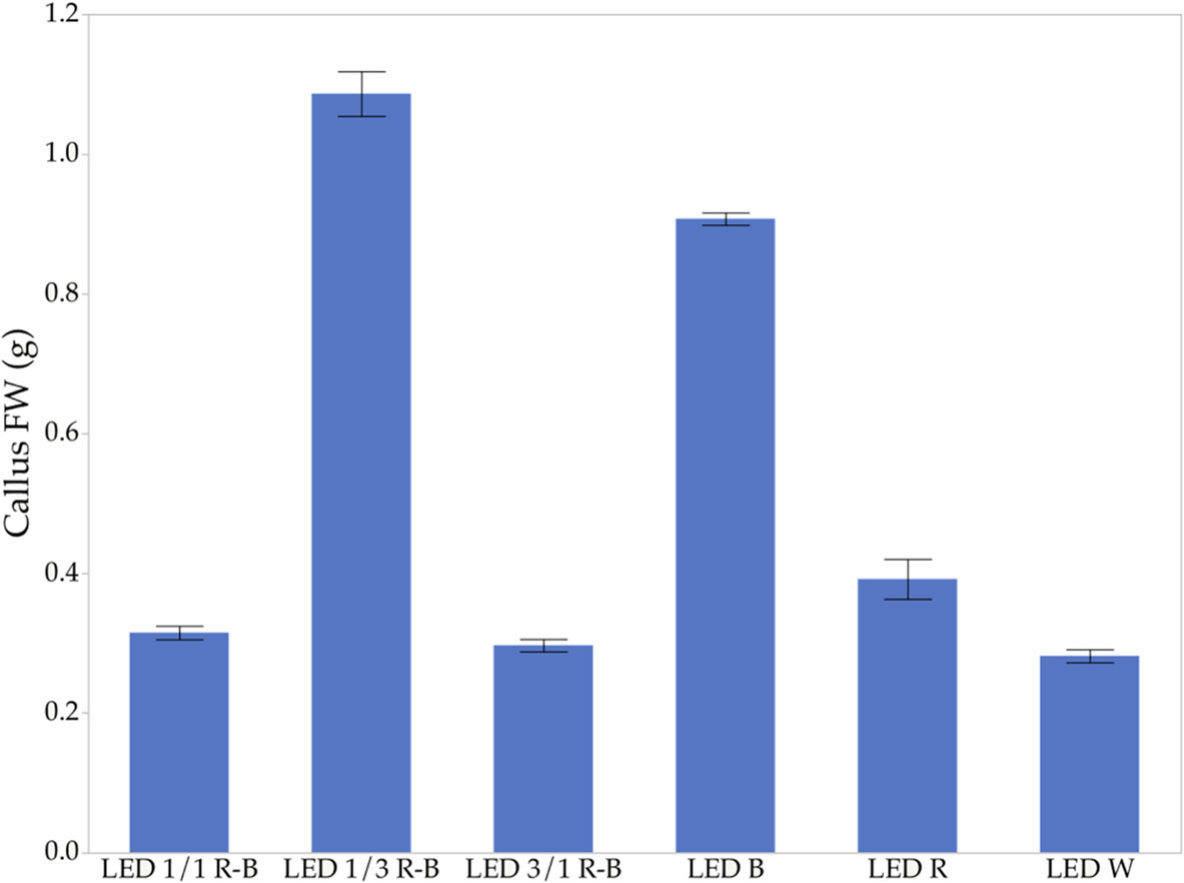
Effect of lights on weight increase of callus

Figure 1 shows the effect of light on the growth of the *C. ensiformis* callus at 30 days, where it is observed that treatments with B have a significant effect on the increase of weight of callus in this plant, having the highest production with the 1/3 R-B combination. While the treatments in which the R is the main one, the increase in callus weight is smaller, being less than 0.35 g in all treatments.

Table 3 shows the statistical analysis regarding the increase of weight in calluses. According to the Tukey test, the combination of 1/3 R-B LED represents an average increase of 1.08 g. In contrast, white light and a combination of 3/1 R and B LED with 0.28, and 0.30 g respectively are below average. These results are engaging in the cultivation of *C. ensiformis*, because the rapid growth of calluses under these conditions helps to have enough biomass for the effects of a scaled process, or even on industrial levels.

**Table 3:** Statistical analysis of the effect on increasing callus weight

Level	Number	Mean	Lower end of 95% CI*	Top end of 95% CI*
LED B	3	0.910 ± 0.021	0.869	0.962
LED R	3	0.403 ± 0.064	0.243	0.563
LED 1/3 R-B	3	1.084 ± 0.078	0.889	1.279
LED 1/1 R-B	3	0.315 ± 0.017	0.273	0.357
LED 3/1 R-B	3	0.296 ± 0.022	0.242	0.351
LED W	3	0.282 ± 0.023	0.225	0.339

*CI: Confidence interval

### Total phenols content

**Fig. 2:**
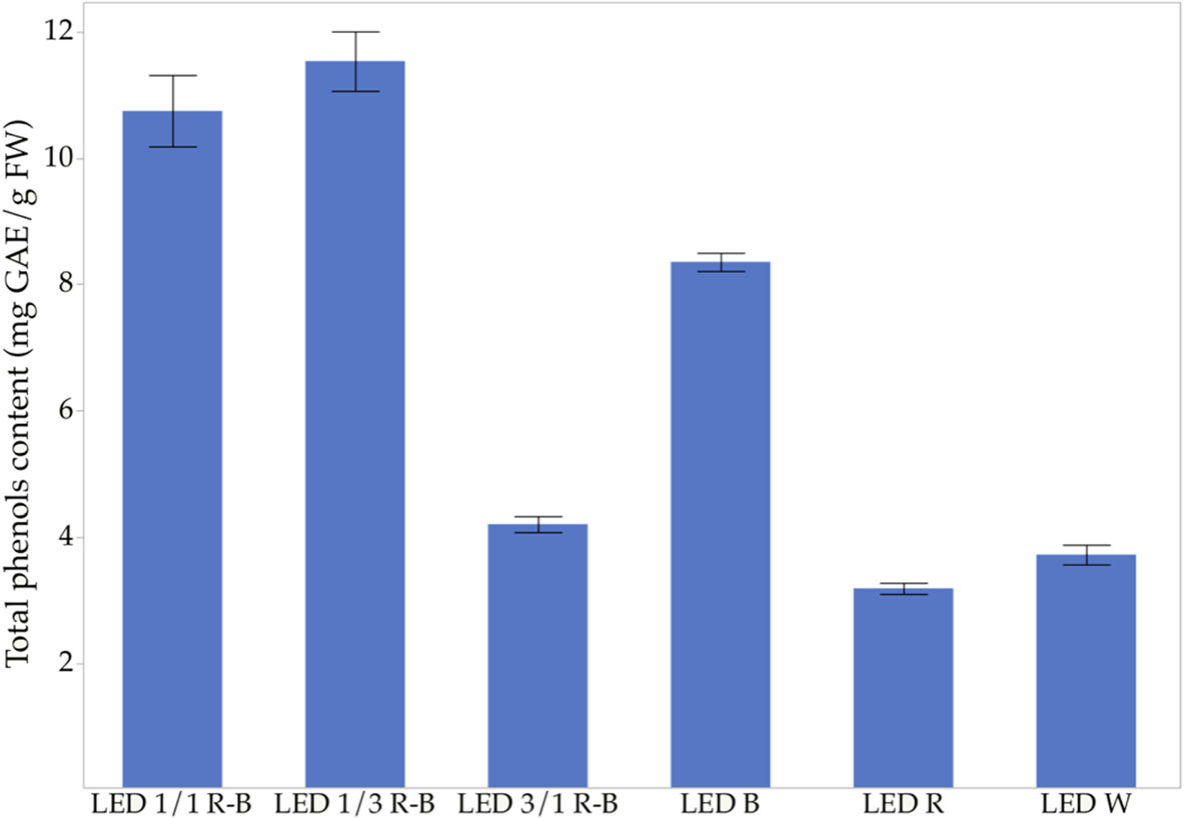
Effect of light on the production of phenolic/polyphenolic compounds

In Fig. 2, the effect of light on the production of phenolic/polyphenolic compounds is shown. It is observed that the production of phenolic compounds increased in all treatments with B, compared to W (white light). In contrast, treatment with R resulted in a much lower mean than that of W. These results may be related to the production of calluses, because the combination of 1/3 R-B LED produced more calluses, results in higher accumulation of total phenolics.

After applying statistical analysis and a Tukey test to the effect of light on the production of phenolic compounds, it was observed that there is a higher production of phenolic compounds when treatment B is in equal or more significant proportion than R. The principal effect of the light occurs in the combined light treatments 1/3 R-B LED and 1/1 R-B LED with an average phenolic production of 11.52 and 10.75 mg GAE/g FW, respectively.

### Chlorophyll a and b content

**Fig. 3:**
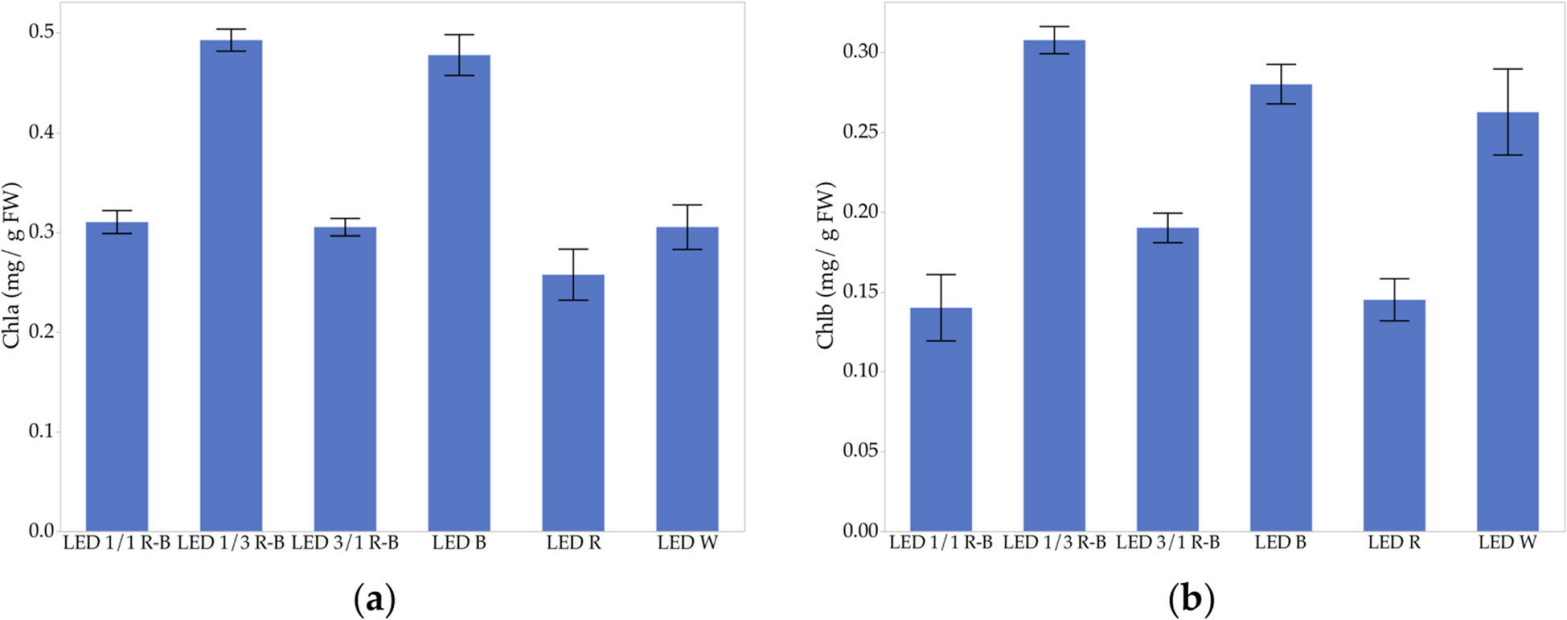
Effect of light on chlorophyll production, (**a**) *Chla* and (**b**) *Chlb*

The effect of light on the production of both *Chla* and *Chlb* is shown in Fig. 3. It is observed that in both treatments, the white light has a production above the average of all the *Chlb* treatments, while the *Chla* is given below the average of all the treatments. However, the highest production is of *Chla*, which reaches an average of 0.32 mg g^− 1^ biomass while the *Chlb* is 0.24 mg g^− 1^ biomass.

The combination of 1/3 R-B LED and the treatment B has higher concentrations of *Chla* and *Chlb* (Fig. 3a and b), this is because it has been widely proven that B favors photosynthesis processes. An interesting effect is observed in the production of *Chla* compared to *Chlb* in the 1/3 R-B and B treatment in which the highest chlorophyll productions occur because *Chla* has a higher production with 0.5 mg g^− 1^ biomass compared to *Chlb* of 0.3 mg g^− 1^ biomass. In the case of *Chlb*, there is a higher standard deviation and a more extensive range. Figure 3 shows that the three best treatments to produce *Chla* and *Chlb* are the combination 1/3 R-B LED, B LED, and W LED. Of the above, the treatment of 1/3 R-B LED is the one with the highest concentration of both chlorophyll a and b with an average of 0.49 and 0.31 mg g^− 1^ biomass, respectively. Moreover, treatment with R, has a lower concentration of chlorophyll both *Chla* and *Chlb*, demonstrating that exposing *C. ensiformis* only to R to produce *Chla* and *Chlb* is not adequate.

### Carotenoids content

**Fig. 4:**
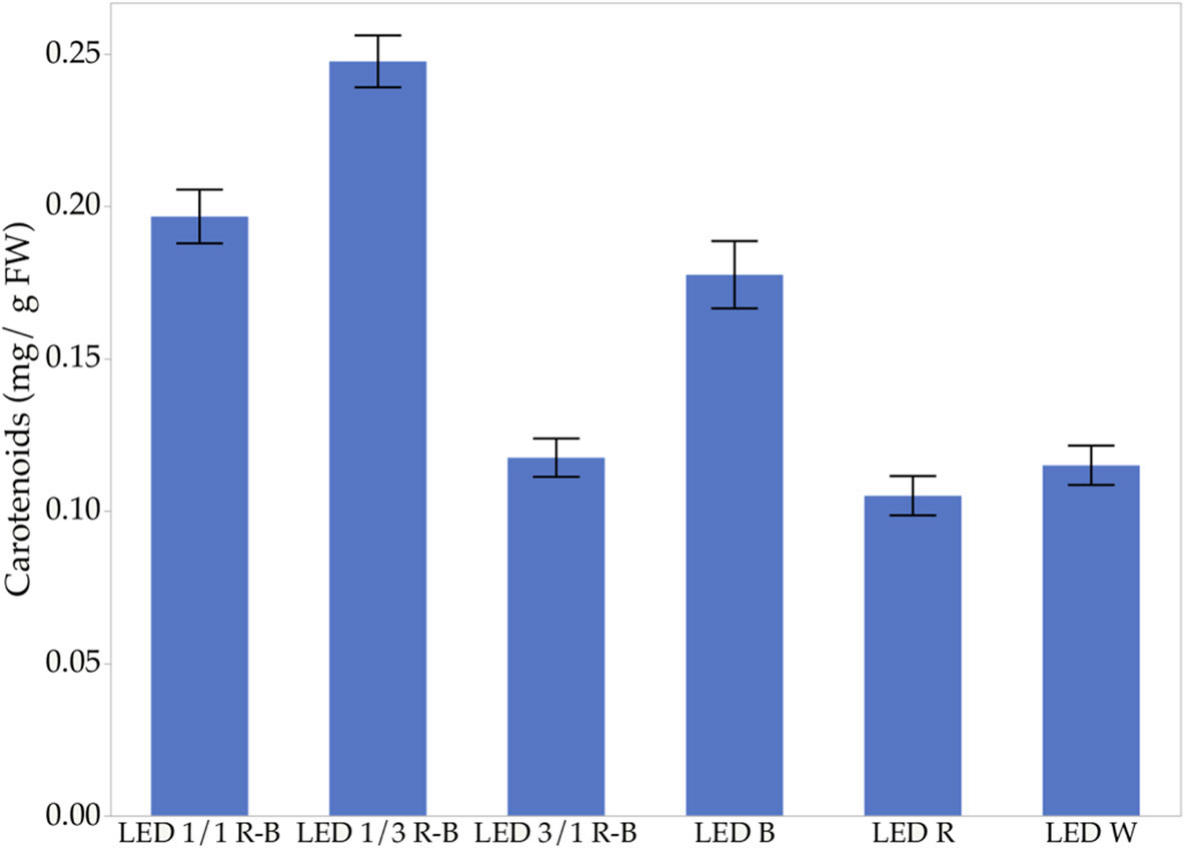
Effect of lights on carotenoid production

Regarding the production of carotenoids, observed in Fig. 4, the R is the one that presents the least number of carotenoids and, again, as in the previous results, it is the light with the lowest production response of bioactive compounds. In the case of the 1/3 R-B combination, it is the best response in the production of carotenoids with an average of 0.25 mg g^− 1^ biomass. This combination is the one that had the best response to the production of compounds of interest and also in the callogenic production, even much greater than that of the control with W. Similarly, only B presented good results, all of them above average in each of the parameters evaluated.

### Antioxidant activity capacity

According to the evaluation carried out on the antioxidant capacity of *C. ensiformis* callus extracts from different light treatments, it could be evidenced that B is the one that has the best effect on this parameter evaluated. Conversely, the R is the one with the lowest activity, with an average of 30.67 μmol TE g^− 1^ FW. The same behavior is observed in the production of phenols and carotenoids concerning the higher antioxidant activity, in which the B in equal or greater quantity than R favors a better production of bioactive compounds (Table 4). Similarly, Table 4 shows that the standard deviation of all treatments is very low compared to the production of *Chla* and *Chlb*.

**Table 4:** Statistical analysis of the effect on antioxidant activity

Treatment	Antioxidant activity (μmol TE g^**− 1**^ FW)	Standard error of the mean	Lower end of 95% CI	Top end of 95% CI
LED B	59.33 ± 0.23	0.33	57.90	60.77
LED R	30.67 ± 0.30	0.33	29.23	32.10
LED 1/3 R-B	65.33 ± 0.13	0.33	63.90	66.77
LED 1/1 R-B	62.33 ± 0.22	0.33	60.90	63.77
LED 3/1 R-B	33.33 ± 0.32	0.33	31.90	34.77
0LED W	40.33 ± 0.33	0.33	38.90	41.77

### Principal component analysis PCA

**Fig. 5:**
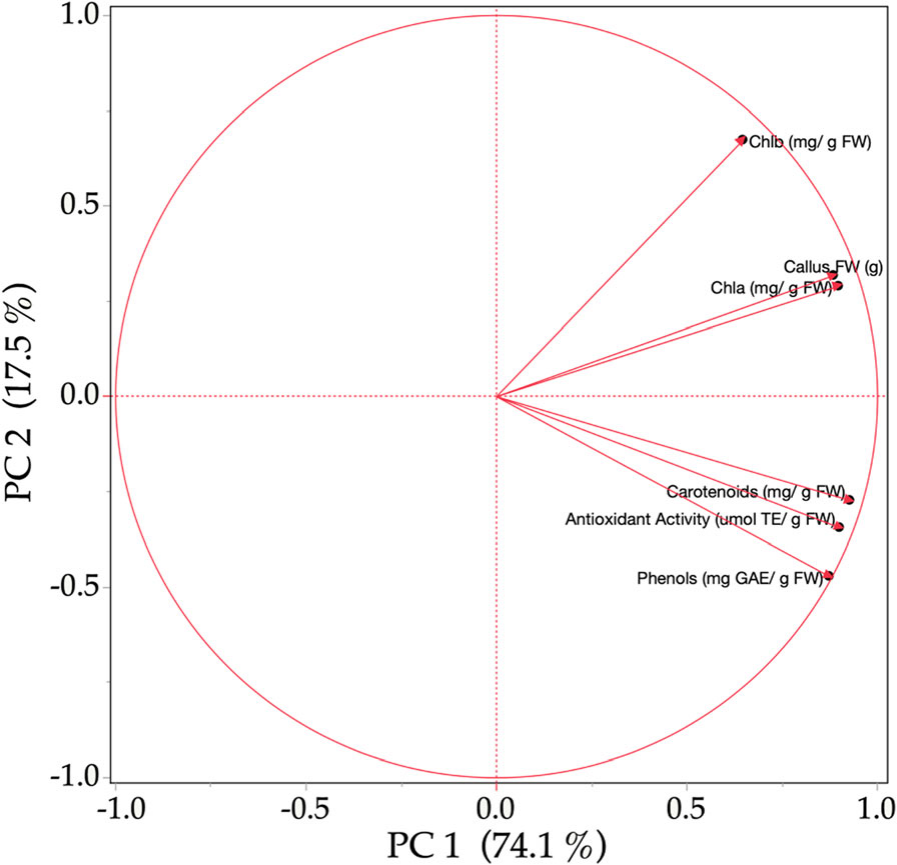
Principal component analysis for the physiological response of *C. ensiformis*

The PCA analysis offers a graphical representation that simplifies the visualization and understanding of data and variables. The analysis shows a relationship between the factors and the different parameters measured. An interaction between *Chla* and *Chlb* is observed, in the same way, there is a correlation between the content of phenols and carotenoids (Fig. 5).

From the PCA analysis, it was found that the first two components explained 92.37% of the overall variation. A high positive correlation was observed between the antioxidant activity and the content of phenols and carotenoids. Callus weight had also shown a positive correlation with the chlorophyll content. The treatments mainly influence this with B and the combination of R and B. Likewise, Table 5 shows the analysis of six principal components showing the percentage of each one.

**Table 5:** Principal components analysis (PCA)

Component	Eigenvalue	Percentage	Cumulative percentage
1	4521	75,359	75,359
2	1021	17,013	92,372
3	0,218	3629	96,001
4	0,150	2499	98,500
5	0,065	1088	99,588
6	0,025	0,412	100,000

## Discussion

It could be verified that the pH is an important factor in the increase of the biomass of *C. ensiformis*, this may be because it affects the intake of minerals, as well as the activity or metabolism of the phytohormones supplied in the medium [35, 36]. In the literature, there is little or no work on the effect of pH on in vitro cultures of *C. ensiformis*, considering that this parameter is essential and that depending on it, the plant will take nutrients better. Its development will also be optimum. Similarly, it has been proven that pH has a profound effect on crop productivity as primary metabolism and biosynthetic pathway enzymes are affected by culture media pH [37–40].

Studies have evaluated on other plants the pH effect on the growth of plants such as tomatoes, *Catharanthus roseus*, *Withania somnifera*, carrot, *Bacopa monnieri*, ectomycorrhizal fungi, among others [37, 41–47]. For this reason, the optimal pH for the in vitro culture of *C. ensiformis* is 5.5. From this, the preliminary production of metabolites such as phenolic compounds and carotenoids were evaluated, as well as antioxidant activity.

In the case of *C. ensiformis* to date, there are few studies on the effect of light on callogenic growth, the production of phenols, chlorophyll a/b, carotenoids, and antioxidant activity. In this study, it was found that B positively affects callus weight, and these results again demonstrate that the light with this wavelength is necessary during plant growth for normal photosynthesis to occur. Similarly, the data accord with other authors who evaluated this light in other plant species [24]. The results of this study show that the considerable increase occurs in a combination of 1/3 R-B LED, and this agrees with other authors who argue that these wavelengths are mostly absorbed by photosynthetic pigments giving an important impact on development [21, 48]. While other authors have found different R-B ratios, in the case of this study (the best 1/3 R-B ratio), which agrees with the results of Li et al. [23] for rapeseed, regarding the biomass increased. Likewise, this behavior is similar to what was argued by Ahmed et al. [49], which says that this type of spectra promotes plant growth, photosynthetic velocity, and biomass accumulation.

Concerning the production of chlorophyll, the same results were exposed as in other studies where it was found that with R the *Chl a/b* production is significantly low compared to B and W [28, 31]. In the case of B, this work found that there is a production equal or higher than the 1/3 R-B LED combination. These results coincide with other studies in which it was found that this favors the production of *Chl a/b* [25–27]. With these results, those argued by other authors are proven that B is essential for the development of chloroplasts, stoma opening, and photomorphogenesis, as well as regulating the biosynthesis of chlorophyll and anthocyanin [50–53]. Also, it was proven with the results that a mixture of lights is necessary for the normal growth of the plant since it favors normal photosynthesis, and besides, the response of the plant can quantitatively resemble those found in the intensity of the radiation [24].

**Fig. 6:**
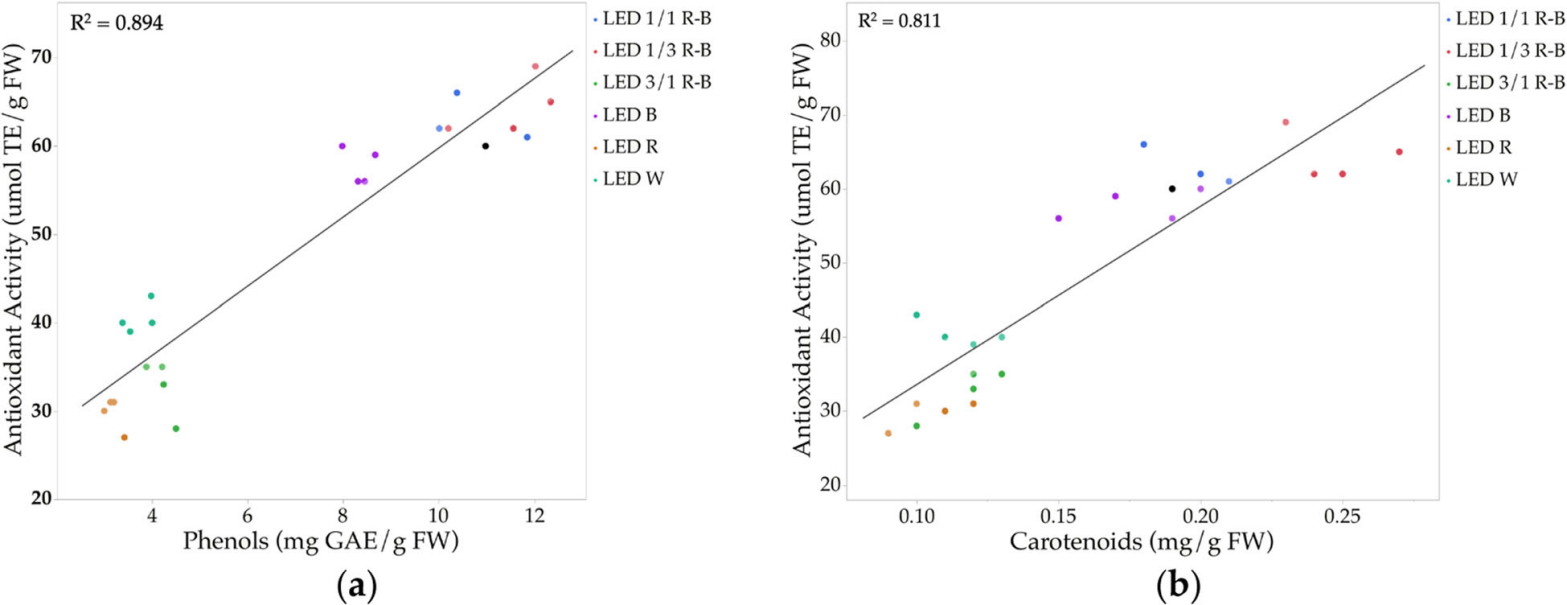
Correlations between antioxidant activity and phenolic and carotenoid compounds (*n* = 18)

*C. ensiformis* shows a significant association between antioxidant capacity, phenols, and carotenoids. These results are similar to those found by Hoffmann et al. [54], which have shown that B illumination favors the potential accumulation of carotenoids. Figure 6 is observed as the correlation between phenolic compounds and the antioxidant capacity, which is directly proportional and accords with other authors who report within the chemical compounds with antioxidant capacity, are the phenolic compounds [55]. Kapoor et al. [10] observed a higher production of phenolic compounds in in vitro cultured corns of the *Rhodiola imbricata* species exposed to B, as well as an increase in the antioxidant capacity of callus extracts. According to the results, these phenolic compounds, that in *C. ensiformis* reach average values of up to 11.52 mg eq. AG/g biomass, intensely contribute to the defense mechanisms against biotic and abiotic stress [56].

Some authors have linked the phenolic content with stress tolerance, either contributing to indirect light protection or participating directly as antioxidants acting as free radical scavengers due to their reducing properties [57, 58]. Also, it has proven that phenolic compounds can act as hydrogen donor agents or singlet oxygen extinguishing electrons and metal chelators [59, 60].

Figure 6 shows the direct connection between the carotenoid and phenolic contents with the antioxidant activity. According to some authors, this happens because compounds have a positive effect on the antioxidants activity [61, 62]. Furthermore, antioxidants are species that can protect organisms from damage caused by oxidative stress induced by free radicals and in this work, it was found that the combination 1/3 R-B LED is the best response to the production of the compounds evaluated [63]. The presence of these antioxidant compounds is important in *C. ensiformis* because it favors the production of vital proteins such as the urease and lectin of the plant. These results demonstrate that in vitro culture with a combination of R and B in 1/3 proportion favors the production of different compounds such as chlorophyll a/b, phenolic contents, and carotenoids that help in antioxidant activity of the plant.

With this work, it was possible to verify that the initial pH in the culture medium is a fundamental factor in giving the proper nutrients to calluses and in the production of compounds of interest such as phenols, carotenoids, and antioxidant activity. Due to that, it has been proven that these are fundamental in the growth of plants and that it affects different structures of it [64, 65]. pH and light are important because they are a significant step for the in vitro production of compounds of importance from this plant, such as urease and lectin. Therefore, it is necessary to carry out new works to investigate how these factors can improve the production of these proteins and how their products could be increased through in vitro cultures.

## Conclusions

It was possible to find the ideal conditions of highest growth under conditions of pH and light of *C. ensiformis*, to evaluate whether under these conditions the production of compounds of interest such as phenolics and carotenoids occurs. In addition to observing the antioxidant activity as a primary factor in the response of the plant to external factors such as the culture media in the callogenic multiplication.

Determining the in vitro culture conditions of *C. ensiformis* represents a valuable instrument for the study and production of important metabolites such as phenols and carotenoids. Its independence from environmental conditions allows for the continuous supply of materials, as well as the use of several strategies to stimulate the specific production of these metabolites. The results of this study show that the pH of the culture medium influences the growth of *C. ensiformis*. In the statistical analysis, it can be seen how the pH of 5.5 shows a better response to callus growth concerning the other four pH evaluated.

The highest production of calluses occurs in the 1/3 R-B LED combined light treatment, which showed a significant increase in biomass, followed by B, while the least effective was with W. However, the R treatment, combination 1/1, and 3/1 R-B LED, show similar behaviors. These results are because the 1/3 RB LED combination is repeated in all the bioactive compounds analyzed in this study, which makes this combination the best treatment for obtaining a large amount of biomass and compounds of interest such as phenols, carotenoids, and chlorophyll a/b.

From this study, it could be demonstrated that *C. ensiformis* not only has a high production of compounds such as urease and lectin but also of compounds such as phenols and carotenoids that are essential for the antioxidant activity of the plant. The in vitro culture of this could be promising to obtain the compounds produced by *C. ensiformis* and it could be of interest at an industrial level as a source of protein.

With these results, the efficiency of B in carotenoid production and photosynthetic activity in *C. ensiformis* was verified. These contrast with the results found by other authors and show once again that the combination of R and B with a higher proportion of B favors a long ratio between oxidizing capacity, phenols, and carotenoids produced in *C. ensiformis*.

## Methods

### Culture conditions

*C. ensiformis* was grown in glass jars (55 cm in diameter by 70 cm high) in the Research Center in Environmental Engineering, Universidad de los Andes, from seeds (Semillas-Camposeeds, Bogotá, Colombia). Callogenic induction and callus propagation were achieved using MS salts and vitamins [66] as a basal medium (Thermo Fisher, New Jersey, USA), and supplemented with 3.0% (w/v) sucrose (Merck, New Jersey, USA)) and 5 g l^− 1^ agar-agar (Merck, New Jersey, USA) callus propagation medium [34].

### pH experiments

To determine the effect of pH on callus growth, these were sowed in MS salts (described Section 2.1). The initial pH of the medium was adjusted with 1 N NaOH (Merck, New Jersey, USA) and 0.1 N HCl (Merck, New Jersey, USA), then they were autoclaved (121 °C, 18 PSI, 20 min). In this work, the pH between 4.5 and 6.0 was used. For the initial weight, a single range was taken, and it is higher than 0.2500 g because it is where the important growth effect was observed for the initial mass. All experiments were performed in quadruplicate. Two hundred eighty-eight glass containers were taken, sterilized at 121 °C, 18 PSI (lbf in^− 2^) for 20 min, weighed empty, and then with the explant, they were immediately cultured at 23 ± 1 °C, with continuous white light for 30 days. After this time, the calluses were weighed again, and the biomass growth over time was obtained by weight difference.

### LED experiments

For LED (*light-emitting diode*) experiments, black painted polypropylene boxes (33 X 52 X 31 cm) were used with red (R; peak at 657 nm), and blue (B; peak at 455 nm) LEDs (ILUMAX, Shenzhen, China). Six light treatments were applied, which were: 100% white light (control) (LED W) and several red (R) to blue (B) ratios, as follows: 100% B, 100% R, 25% R and 75% B (1/3 R-B), 75% R and 25% B (3/1 R-B), and 50% R and 50% B (1/1 R-B), and LED W. In each treatment, three glass containers with 25 ml of MS salts containing 0.25 g of fresh callus were seeded. Calluses were grown for 60 days, until the callogenic mass increased and exposed to light treatments for 30 days. The laboratory conditions were a relative humidity of 52%, with an average temperature of 20/16 °C (light/day), the plants were exposed to a 12 h/12 h photoperiod. Then, callus biomass was analyzed and used for the determination of total phenolics, chlorophyll, carotenoids, and antioxidant activity.

### Total phenolics

The content of total phenolic in callus extract was determined by the Folin-Ciocalteu method [67]. Fresh calluses (200 mg) were extracted with methanol using a Soxhlet apparatus. 1 mL of methanolic callus extract was mixed with 5 ml of Folin-Ciocalteu reagent (Sigma-Aldrich, St. Louis, United States) (diluted 10-fold) and 4 ml of sodium carbonate solution (7500 mg l^− 1^). Then, the absorbance at 765 nm was measured after 1 h. The calibration curve was prepared with methanolic gallic acid solutions, which were mixed with the same reagents described above, and after 1 h the absorbance was measured. Total phenolic content in callus methanolic extracts was expressed in Gallic Acid Equivalents (GAE) by the equation:

1 $$ TP=\frac{\left(c\ast V\right)}{FW} $$

Where, TP is the total content of phenolic compounds expressed like mg GAE g^− 1^ FW, c is the concentration of gallic acid deduced from the calibration curve (mg ml^− 1^), V is extract volume (ml), and FW is the weight of the fresh callus (g).

### Chlorophyll and carotenoids determination

The content of chlorophyll and carotenoids was quantified in acetone extract. In other words, 100 mg of fresh callus were crushed in 5 ml of chilled acetone (80% v/v). Then, the extract was centrifuged at 2500 rpm for 5 min, and absorbance of the supernatant was read at 660, 645, and 470 nm. The content of *Chla*, *Chlb,* and carotenoids was calculated in mg g^− 1^ FW biomass using the following equations, according to Wellburn [68].

2 $$ CHla=\left\{\frac{\left[12.21\left({A}_{660}\right)-2.81\left({A}_{645}\right)\right]V}{\left(1000\ast FW\right)}\right\} $$

3 $$ CHlb=\left\{\frac{\left[20.13\left({A}_{645}\right)-5.03\left({A}_{660}\right)\right]V}{\left(1000\ast FW\right)}\right\} $$

4 $$ Carotenoid=\left\{\frac{\left[1000\left({A}_{470}\right)-3.27(CHla)-104\left(\mathrm{C} Hlb\right)\right]V}{\left(1000\ast FW\right)}\right\} $$

Where A_660_, A_645_, and A_470_ are the value of absorbance in nm, V is the extract volume, and FW is the weight of the fresh callus.

### Antioxidant activity capacity

The antioxidant activity of callus methanolic extract was determined by 2,7′- dichlorodihydrofluorescein diacetate (DCFH) probe, which reacts indiscriminately with reactive oxygen species (ROS) and reactive nitrogen species (RNS) generated by the compound azo, 2,2′-diazobis (2-amidinopropane dihydrochloride) (AAPH) in an aqueous medium and forms the fluorescent compound 2,7-dichlorofluorescein (DCF). The antioxidants in the samples capture ROS and RNS and reduce the fluorescence emitted by DCF. 50 μl of a 0.3 M AAPH solution, 50 μl of a 2.4 mM DCFH ethanolic solution, 2850 μl of 75 mM phosphate buffer (pH 7.4), and 50 μl of the methanolic callus extract (described Section 2.4), which was obtained by mixing 0.3 g of macerated fresh callus using liquid nitrogen and mortar, with 2 ml of 10 mM phosphate buffer (pH 7.0). The intensity of fluorescence emitted during the first 10 min was read and compared with the intensity emitted in the absence of the sample (λ excitation: 326 nm, a λ emission: 432 nm and 10 nm slit). The results are expressed as mg μmol of Trolox Equivalents (TE) per g of fresh callus biomass by constructing a standard curve using different concentrations of TROLOX® [69].

### Statistical analysis

All statistical analysis were performed using two softwares. Statgraphics 18 centurion software was used for the analysis of the effect of pH on callus growth. For the analysis of the effect of light on the growth of calluses and the effect of the production of bioactive substances, the software JMP-Pro version 13.1.0 was used. All experiments were performed in quadruplicate.

## Supplementary information

**Additional file 1.**

**Additional file 2.**

## Data Availability

All data generated and analyzed during this study are included in the published article.

## Abbreviations

A_660_, A_645_, A_470_: Value of absorbance (nm)
AAPH: Azo, 2,2′-diazobis (2-amidinopropane dihydrochloride)
ANOVA: Analysis of variance
B: Blue light
C: Concentration of gallic acid (mg ml^− 1^)
*Chla*: Chlorophyll a
*Chlb*: Chlorophyll b
CI: Confidence interval
DCF: 2,7-dichlorofluorescein
DCFH: 2,7′- dichlorodihydrofluorescein diacetate
df: *Degrees of freedom*
FW: Weight of the fresh callus (g)
GAE: Gallic Acid Equivalents
PCA: Principal component analysis
PFR: Far-red light
R: Red light
RNS: Reactive nitrogen species
ROS: Reactive oxygen species
TE: Trolox Equivalents
TP: Total content of phenolic compounds expressed like mg (GAE g^− 1^ FW)
V: Extract volume (ml)
W: White light
