# Supplementary material for: Preliminary study of the production of metabolites from in vitro cultures of C. ensiformis
Source: BMC Biotechnol. 2020 Sep 10;20:49. doi: 10.1186/s12896-020-00642-x (PMC7488093; doi:10.1186/s12896-020-00642-x)
Supplement: Supplementary file 1 — Additional file 1. [file 12896_2020_642_MOESM1_ESM.docx]

Preliminary study of the production of metabolites from in vitro cultures of *C. ensiformis*

Juan F. Saldarriaga ^1,^*, Yuby Cruz ^1^ and Julián E. López ^2^

^1^ Dept. of Civil and Environmental Engineering, Universidad de los Andes, Carrera 1Este #19A-40, Bogotá, Colombia 111711.

^2^ Dept. of Environmental Engineering, Universidad de Medellín, Carrera 87 #30-65, Medellín, Colombia 050026.

*****jf.saldarriaga@uniandes.edu.co, juanfelorza@gmail.com

1. **Additional file 1**

Below are all the statistical analyzes performed that are not shown in the manuscript

A.1. Effect of light on the increase in callus weight


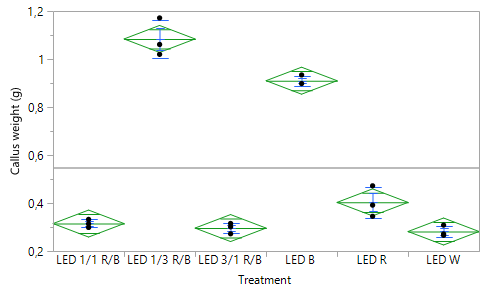


Figure A.1. Effect of light treatment on increasing callus weight

Table A.1. Analysis of variance (ANOVA) for the effect of light on biomass increase

| **Level** | **Number** | **Mean** | **Standard deviations** | **Standard error of the mean** | **Lower end of 95% CI** | **Top end of 95% CI** |
| --- | --- | --- | --- | --- | --- | --- |
| LED B | 4 | 0.910 | 0.021 | 0.012 | 0.859 | 0.962 |
| LED R | 4 | 0.403 | 0.064 | 0.037 | 0.243 | 0.563 |
| LED 1/3 R/B | 4 | 1.084 | 0.078 | 0.045 | 0.890 | 1.279 |
| LED 1/1 R/B | 4 | 0.315 | 0.017 | 0.010 | 0.273 | 0.367 |
| LES 3/1 R/B | 4 | 0.296 | 0.022 | 0.013 | 0.242 | 0.351 |
| LED W | 4 | 0.282 | 0.023 | 0.013 | 0.224 | 0.339 |

Table A.2. Tukey analysis for the effect of the light on biomass increase

| **Level** |  |  |  | **Mean** |
| --- | --- | --- | --- | --- |
| LED 1/3 R/B | A |  |  | 1.084 |
| LED B |  | B |  | 0.910 |
| LED R |  |  | C | 0.403 |
| LED 1/1 R/B |  |  | C | 0.315 |
| LED 3/1 R/B |  |  | C | 0.296 |
| LED W |  |  | C | 0.282 |

A.2. Statistical analysis for the content of total phenols


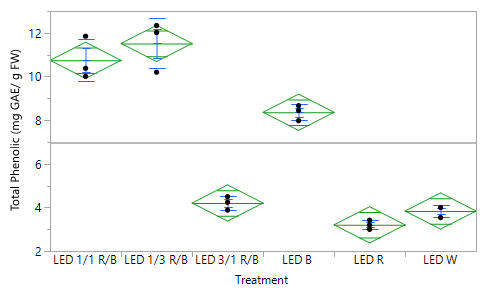


Figure A.2. Effect of light on the production of total phenolic compounds

Table A.3. Analysis of variance (ANOVA) for the production of total phenolic compounds

| **Level** | **Number** | **Mean** | **Standard deviations** | **Standard error of the mean** | **Lower end of 95% CI** | **Top end of 95% CI** |
| --- | --- | --- | --- | --- | --- | --- |
| LED B | 4 | 8.367 | 0.352 | 0.203 | 7.491 | 9.242 |
| LED R | 4 | 3.207 | 0.210 | 0.121 | 2.685 | 3.729 |
| LED 1/3 R/B | 4 | 11.520 | 1.154 | 0.666 | 8.653 | 14.387 |
| LED 1/1 R/B | 4 | 10.747 | 0.973 | 0.562 | 8.329 | 13.164 |
| LES 3/1 R/B | 4 | 4.207 | 0.311 | 0.180 | 3.433 | 4.980 |
| LED W | 4 | 3.840 | 0.260 | 0.150 | 3.194 | 4.486 |

Table A.4. Tukey analysis for the production of total phenolic compounds

| **Level** |  |  |  | **Mean** |
| --- | --- | --- | --- | --- |
| LED 1/3 R/B | A |  |  | 11.520 |
| LED 1/1 R/B | A |  |  | 10.747 |
| LED B |  | B |  | 8.367 |
| LED 3/1 R/B |  |  | C | 4.207 |
| LED W |  |  | C | 3.840 |
| LED R |  |  | C | 3.207 |

A.3. Statistical analysis for the chlorophyll a production


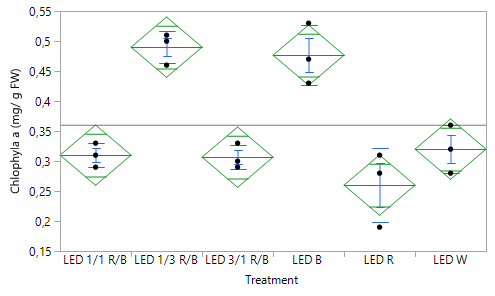


Figure A.3. Effect of light on the production of chlorophyll a

Table A.5. Analysis of variance (ANOVA) for the production of chlorophyll a

| **Level** | **Number** | **Mean** | **Standard deviations** | **Standard error of the mean** | **Lower end of 95% CI** | **Top end of 95% CI** |
| --- | --- | --- | --- | --- | --- | --- |
| LED B | 4 | 0.477 | 0.050 | 0.029 | 0.352 | 0.602 |
| LED R | 4 | 0.260 | 0.062 | 0.036 | 0.105 | 0.415 |
| LED 1/3 R/B | 4 | 0.490 | 0.026 | 0.015 | 0.424 | 0.556 |
| LED 1/1 R/B | 4 | 0.310 | 0.020 | 0.011 | 0.260 | 0.360 |
| LES 3/1 R/B | 4 | 0.307 | 0.021 | 0.012 | 0.255 | 0.358 |
| LED W | 4 | 0.320 | 0.040 | 0.023 | 0.221 | 0.419 |

Table A.6. Tukey analysis for the production of chlorophyll a

| **Level** |  |  | **Mean** |
| --- | --- | --- | --- |
| LED 1/3 R/B | A |  | 0.490 |
| LED B | A |  | 0.477 |
| LED W |  | B | 0.320 |
| LED 1/1 R/B |  | B | 0.310 |
| LED 3/1 R/B |  | B | 0.307 |
| LED R |  | B | 0.260 |

A.4. Statistical analysis for the chlorophyll a production


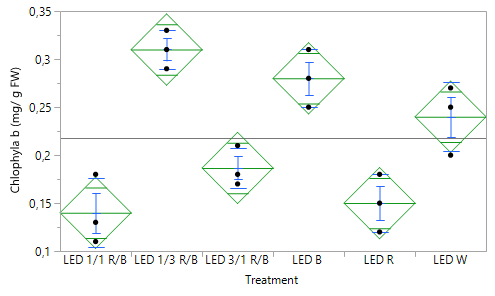


Figure A.4. Effect of light on the production of chlorophyll b

Table A.7. Analysis of variance (ANOVA) for the production of chlorophyll a

| **Level** | **Number** | **Mean** | **Standard deviations** | **Standard error of the mean** | **Lower end of 95% CI** | **Top end of 95% CI** |
| --- | --- | --- | --- | --- | --- | --- |
| LED B | 4 | 0.280 | 0.030 | 0.017 | 0.205 | 0.354 |
| LED R | 4 | 0.150 | 0.030 | 0.017 | 0.075 | 0.224 |
| LED 1/3 R/B | 4 | 0.310 | 0.020 | 0.012 | 0.260 | 0.360 |
| LED 1/1 R/B | 4 | 0.140 | 0.036 | 0.021 | 0.050 | 0.230 |
| LES 3/1 R/B | 4 | 0.187 | 0.021 | 0.012 | 0.135 | 0.238 |
| LED W | 4 | 0.240 | 0.036 | 0.021 | 0.150 | 0.330 |

Table A.8. Tukey analysis for the production of chlorophyll a

| **Level** |  |  |  | **Mean** |
| --- | --- | --- | --- | --- |
| LED 1/3 R/B | A |  |  | 0.310 |
| LED B | A |  |  | 0.280 |
| LED W | A | B |  | 0.240 |
| LED 3/1 R/B |  | B | C | 0.187 |
| LED R |  |  | C | 0.150 |
| LED 1/1 R/B |  |  | C | 0.140 |

A.5. Statistical analysis to determine the effect of antioxidant capacity


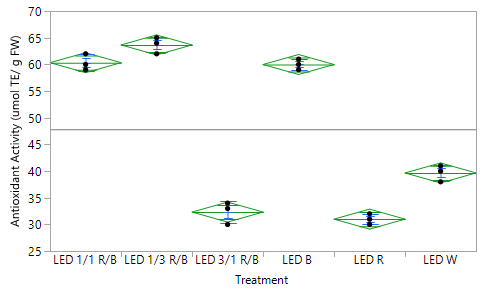


Figure A.5. Effect of light on the antioxidant activity

Table A.9. Analysis of variance (ANOVA) for the effect of antioxidant activity

| **Level** | **Number** | **Mean** | **Standard deviations** | **Standard error of the mean** | **Lower end of 95% CI** | **Top end of 95% CI** |
| --- | --- | --- | --- | --- | --- | --- |
| LED B | 4 | 5.933 | 0.0577 | 0.033 | 5.790 | 6.077 |
| LED R | 4 | 3.067 | 0.0577 | 0.033 | 2.923 | 3.219 |
| LED 1/3 R/B | 4 | 6.533 | 0.0577 | 0.033 | 6.390 | 6.677 |
| LED 1/1 R/B | 4 | 6.233 | 0.0577 | 0.033 | 6.090 | 6.377 |
| LES 3/1 R/B | 4 | 3.333 | 0.0577 | 0.033 | 3.190 | 3.477 |
| LED W | 4 | 4.033 | 0.0577 | 0.033 | 3.890 | 4.177 |

Table A.10. Tukey analysis for the effect of antioxidant activity

| **Level** |  |  |  |  |  |  | **Mean** |
| --- | --- | --- | --- | --- | --- | --- | --- |
| LED 1/3 R/B | A |  |  |  |  |  | 6.533 |
| LED 1/1 R/B |  | B |  |  |  |  | 6.233 |
| LED B |  |  | C |  |  |  | 5.933 |
| LED W |  |  |  | D |  |  | 4.033 |
| LED 3/1 R/B |  |  |  |  | E |  | 3.333 |
| LED R |  |  |  |  |  | F | 3.067 |

A.6. effect of light on carotenoid content


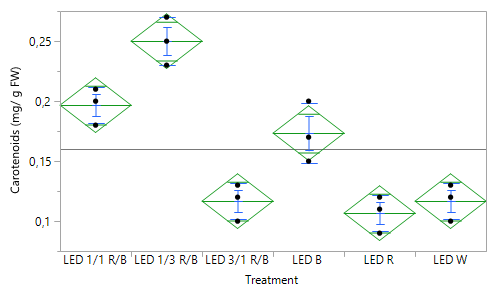


Figure A.6. Effect of light on the carotenoid content

Table A.11. Analysis of variance (ANOVA) for the effect of carotenoid content

| **Level** | **Number** | **Mean** | **Standard deviations** | **Standard error of the mean** | **Lower end of 95% CI** | **Top end of 95% CI** |
| --- | --- | --- | --- | --- | --- | --- |
| LED B | 4 | 0.173 | 0.025 | 0.014 | 0.111 | 0.236 |
| LED R | 4 | 0.107 | 0.015 | 0.009 | 0.069 | 0.145 |
| LED 1/3 R/B | 4 | 0.250 | 0.020 | 0.011 | 0.200 | 0.300 |
| LED 1/1 R/B | 4 | 0.197 | 0.015 | 0.009 | 0.159 | 0.235 |
| LES 3/1 R/B | 4 | 0.117 | 0.015 | 0.009 | 0.079 | 0.155 |
| LED W | 4 | 0.117 | 0.015 | 0.009 | 0.079 | 0.155 |

Table A.12. Tukey analysis for the effect of carotenoid content

| **Level** |  |  |  | **Mean** |
| --- | --- | --- | --- | --- |
| LED 1/3 R/B | A |  |  | 0.250 |
| LED 1/1 R/B |  | B |  | 0.197 |
| LED B |  | B |  | 0.173 |
| LED 3/1 R/B |  |  | C | 0.117 |
| LED W |  |  | C | 0.117 |
| LED R |  |  | C | 0.107 |

A.7. Correlations for total phenols with respect to antioxidant capacity


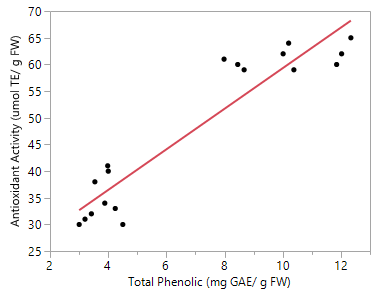


Figure A.7. Parity graph for the correlation between total phenols and antioxidant capacity

Table A.13. Fitting of the correlations between total phenols and antioxidant capacity

| **Variable** | **Value** |
| --- | --- |
| R^2^ | 0.914 |
| Root of the root mean square error | 0.440 |
| Average response | 4.856 |
| Observations (or sum of weights) | 18.000 |

A.8. Correlations for carotenoid content with respect to antioxidant capacity


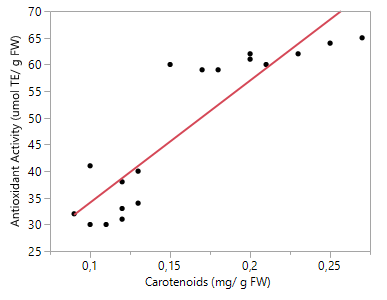


Figure A.8. Parity graph for the correlation between carotenoid content and antioxidant capacity

Table A.14. Fitting of the correlations between carotenoid content and antioxidant capacity

| **Variable** | **Value** |
| --- | --- |
| R^2^ | 0.807 |
| Root of the root mean square error | 0.661 |
| Average response | 4.820 |
| Observations (or sum of weights) | 18.000 |

A.9. Principal components analysis (PCA) showing the relationship between biomass and chlorophyll. and the relationship between phenols and carotenoids with antioxidant capacity.


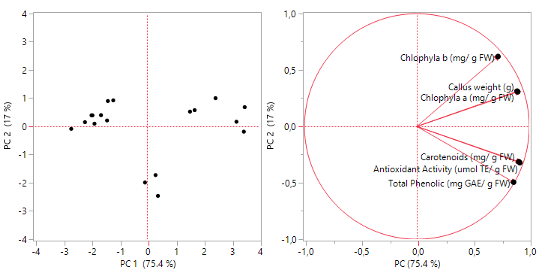


Table A.15. Principal component analysis and parity correlation

| **Variable** | **by variable** | **Correlation** | **Count** | **Lower end of 95% CI** | **Top end of 95% CI** | **Significance test** |
| --- | --- | --- | --- | --- | --- | --- |
| Phenols (mg eq AG/g biomass) | callus weight (g) | 0.612 | 24 | 0.203 | 0.839 | 0.007 |
| Chlorophyll a (mg/g biomass) | callus weight (g) | 0.887 | 24 | 0.717 | 0.957 | 0.0001 |
| Chlorophyll a (mg/g biomass) | Phenols (mg eq AG/  g biomass) | 0.629 | 24 | 0.230 | 0.847 | 0.005 |
| Chlorophyll b (mg/g biomass) | Callus weight (g) | 0.767 | 24 | 0.467 | 0.908 | 0.000 |
| Chlorophyll b (mg/g biomass) | Phenols (mg eq AG/g biomass) | 0.312 | 24 | -0.181 | 0.680 | 0.207 |
| Chlorophyll b (mg/g biomass) | Callus weight (g) | 0.774 | 24 | 0.481 | 0.912 | 0.000 |
| Carotenoids (mg/g biomass) | callus weight (g) | 0.703 | 24 | 0.352 | 0.881 | 0.001 |
| Carotenoids (mg/g biomass) | Phenols (mg eq AG/g biomass) | 0.920 | 24 | 0.795 | 0.970 | 0.000 |
| Carotenoids (mg/g biomass) | Chlorophyll a (mg/g biomass) | 0.663 | 24 | 0.284 | 0.863 | 0.003 |
| Carotenoids (mg/g biomass) | Chlorophyll b (mg/g biomass) | 0.501 | 24 | 0.045 | 0.784 | 0.034 |
| Antioxidant | callus weight (g) | 0.678 | 24 | 0.309 | 0.870 | 0.002 |
| Antioxidant | Phenols (mg eq AG/g biomass) | 0.950 | 24 | 0.868 | 0.981 | 0.000 |
| Antioxidant | Chlorophyll a (mg/g biomass) | 0.720 | 24 | 0.382 | 0.888 | 0.001 |
| Antioxidant | Chlorophyll b (mg/g biomass) | 0.482 | 24 | 0.019 | 0.774 | 0.043 |
| Antioxidant | Carotenoids (mg/g biomass) | 0.899 | 24 | 0.744 | 0.962 | 0.000 |
